# Novel variants in *COL4A4* and *COL4A5* are rare causes of FSGS in two unrelated families

**DOI:** 10.1038/s41439-018-0016-8

**Published:** 2018-07-10

**Authors:** Stephanie L. Hines, Anjali Agarwal, Mohamedanwar Ghandour, Nabeel Aslam, Ahmed N. Mohammad, Paldeep S. Atwal

**Affiliations:** 1 Department of Internal Medicine, Mayo Clinic, Jacksonville, FL 32224 USA; 2 Department of Clinical Genomics, Mayo Clinic, Jacksonville, FL 32224 USA; 3 Department of Nephrology, Mayo Clinic, Jacksonville, FL 32224 USA

## Abstract

We report two female patients with focal segmental glomerulosclerosis and chronic kidney disease. The first patient was found to have a heterozygous, de novo, pathogenic variant in *COL4A5* (c.141+1G>A, IVS2+1G>A), which is associated with Alport syndrome. The second patient was found to have a heterozygous, likely pathogenic variant in *COL4A4* (c.2842G>T). Both these variants in *COL4A5* and *COL4A4* are novel, and they were detected using whole exome sequencing and gene panel testing, respectively. Additionally, we discuss the complexities of diagnosis in such cases and the benefits of using the abovementioned diagnostic approaches.

## Kidney dysfunction: Identifying mutations linked with focal segmental glomerulosclerosis

Researchers in the USA have identified two mutations linked with unrelated cases of the kidney disease focal segmental glomerulosclerosis (FSGS) and compared different diagnostic techniques. This disease is involved in roughly 40% of adult kidney dysfunction. Using sequencing of all protein-coding genes, Paldeep Atwal’s team at the Mayo Clinic, Jacksonville, identified a novel mutation in an FSGS patient. The mutation was not inherited, indicating that the patient’s siblings are not at increased risk. There were also mutations in other genes which may account for the patient’s vision loss. The team tested a second FSGS patient by screening kidney-related genes specifically, revealing a novel mutation in a different gene. Although the targeted approach is cheaper and quicker, these findings highlight the diagnostic benefits of full sequencing, which can reveal relevant mutations in unexpected genes.

## Introduction

Focal segmental glomerulosclerosis (FSGS) is a progressive kidney disease that can be either a primary renal disorder or secondary due to other etiologies, including genetic etiologies^1^. It is the culprit of approximately 40% of nephrotic syndrome cases in adults and 20% in children^2, 3^. As the name suggests, FSGS is characterized by focal [less than 50% of glomeruli are affected in light microscopy (LM)] and segmental [less than 50% of a glomerular tuft is affected] glomerular sclerosis and by varying degrees of foot process effacement^2^. Pathogenic variants in genes that are specific to the function and structure of podocytes, such as *TRPC6*, *ACTN4*, *WT1*, *CD2AP*, *INF2*, *NPHS2*, and *PLCE1*, have been reported to cause familial FSGS^4^. Pathogenic variants in collagen type IV genes (*COL4A3, COL4A4*, and *COL4A5*) are related to a spectrum of disorders with heterogeneous clinical manifestations.

Alport syndrome (AS), which is caused by glomerular membrane defects, has X-linked and autosomal dominant/recessive modes of inheritance. Classical AS has X-linked inheritance due to pathogenic variants in *COL4A5* (86% of cases) and usually presents in early childhood with microscopic or gross hematuria, which progresses to end stage renal disease^5^.The remaining 15% of AS has autosomal inheritance and is thought to be due to pathogenic variants in the *COL4A3* and *COL4A4* genes. Thin basement membrane nephropathy (TBMN), which is also characterized by glomerular membrane abnormalities, has been linked to *COL4A3* and *COL4A4* as well^6^. Renal disorders causing glomerular basement membrane structural abnormalities are known collectively as collagen IV nephropathies (COL4Ns)^7^. FSGS typically presents with proteinuria, often in the nephrotic range, which progresses over months to years to a reduction in glomerular filtration rate (GFR). *COL4A3* and *COL4A4*, which are located on chromosome 2, have been associated with FSGS^8^. Herein, we present two families with novel COL4A4 and COL4A5 variants diagnosed by massively parallel exome sequencing and discuss the complexities of managing these genetic results.

## Materials and methods

To identify the molecular bases of the patients’ phenotypes, whole exome sequencing was utilized in the first patient, while the second proband was tested with a nephrotic syndrome/focal segmental glomerulosclerosis sequencing panel.

## Results

The exome sequencing revealed a heterozygous, de novo, pathogenic variant in *COL4A5* (c.141+1G>A, IVS2+1G>A), a heterozygous, paternally inherited, pathogenic variant in *ABCA4* (c.2588G>C, p.G863A) and a heterozygous, maternally inherited, likely pathogenic variant in *ABCA4* (c.203C>G, p.P68R). The nephrotic syndrome/focal segmental glomerulosclerosis sequencing panel detected a heterozygous, likely pathogenic variant in *COL4A4* (c.2842G>T).

### Family 1

**Fig. 1:**
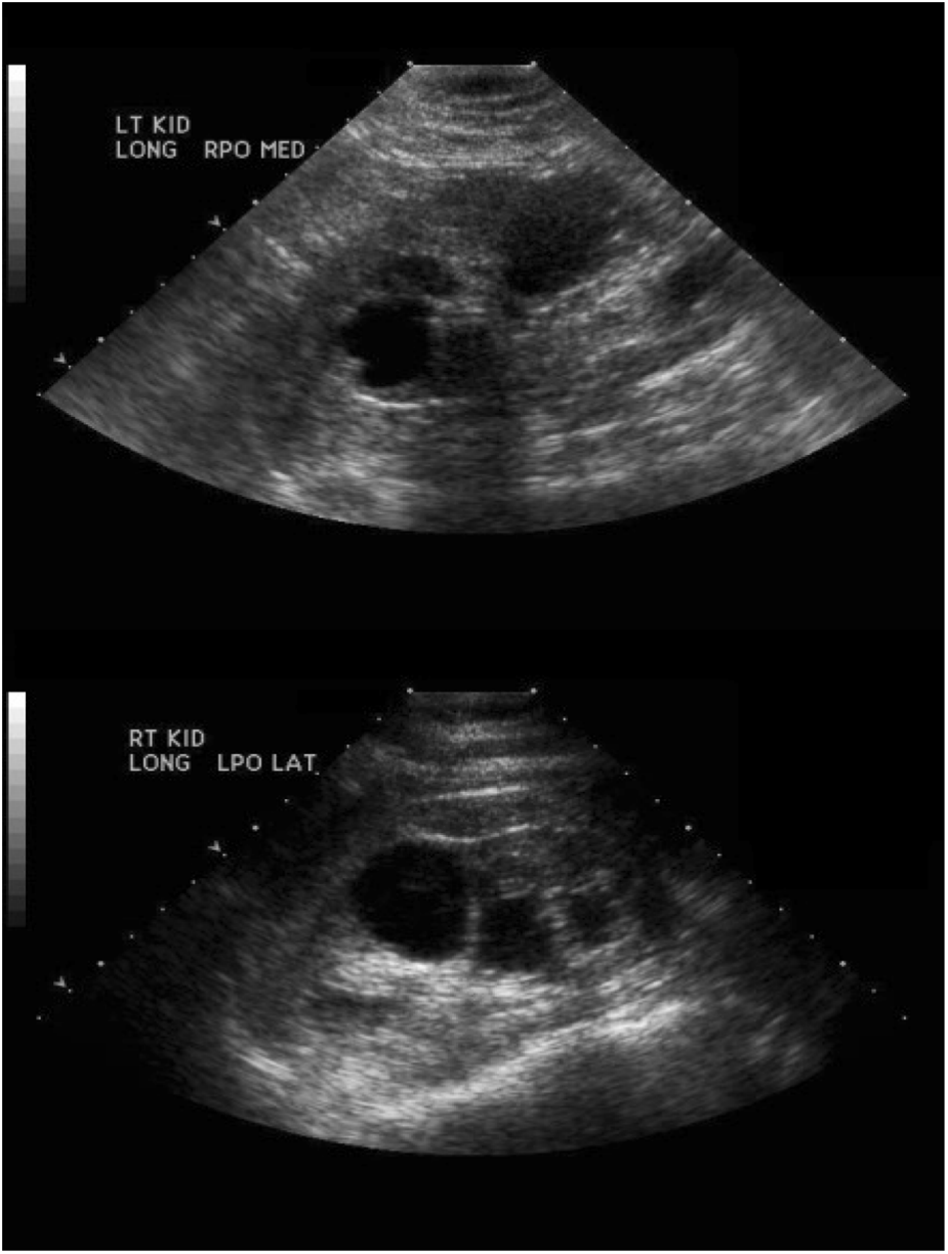
Renal ultrasound showing multiple bilateral cysts

Our first proband is a 52-year-old female with stage IV chronic kidney disease (estimated GFR of 28.2 mL/min), microalbuminuria (microalbumin/creatinine ratio of 206 mg/g) and microscopic hematuria. Her kidney disease first started at age 7, when she presented with microscopic hematuria, which was thought to be benign. At that point in time, her kidney function was normal, and she subsequently did well until age 34, when she developed proteinuria (3 grams of protein/24-h urine), hypertension and creatinine clearance of 45 mL/min. She underwent a renal biopsy, which showed FSGS with no foot process effacement, suggestive of a secondary form. She was started on 30 mg prednisone daily, which resulted in remission of her proteinuria. A renal ultrasound scan showed multiple bilateral renal cysts (Fig. 1), though no family history of ADPKD was found. However, her son was also diagnosed with microscopic hematuria at age 7. In addition to the kidney disease, the patient has chronic arthritis, for which she takes low-dose prednisone; hypermobile joints; migraine headaches; and macular degeneration, which was diagnosed at age 40. She underwent a brain MRI to evaluate the headaches, and the result was normal except for a mild Chiari malformation. A genetic etiology to her kidney disease was suspected, and samples from the proband’s mother, father and affected son were submitted for variant segregation analysis by whole exome sequencing (WES). The patient was found to have a heterozygous, de novo, pathogenic variant in *COL4A5* (c.141+1G>A, IVS2+1G>A), which is associated with Alport syndrome. As it is de novo, her siblings are unlikely to be at risk for the disorder, although germline mosaicism cannot be excluded. Her son (18 years of age) carries this variant, giving him the genetic diagnosis of Alport syndrome as well. Moreover, the patient was found to be a compound heterozygote for ABCA4 variants. She has a heterozygous, paternally inherited, pathogenic variant in *ABCA4* (c.2588G>C, p.G863A) and a heterozygous, maternally inherited, likely pathogenic variant in *ABCA4* (c.203 C >G, p.P68R). These variants likely are the cause for the patient’s macular degeneration and vision loss.

### Family 2

**Fig. 2:**
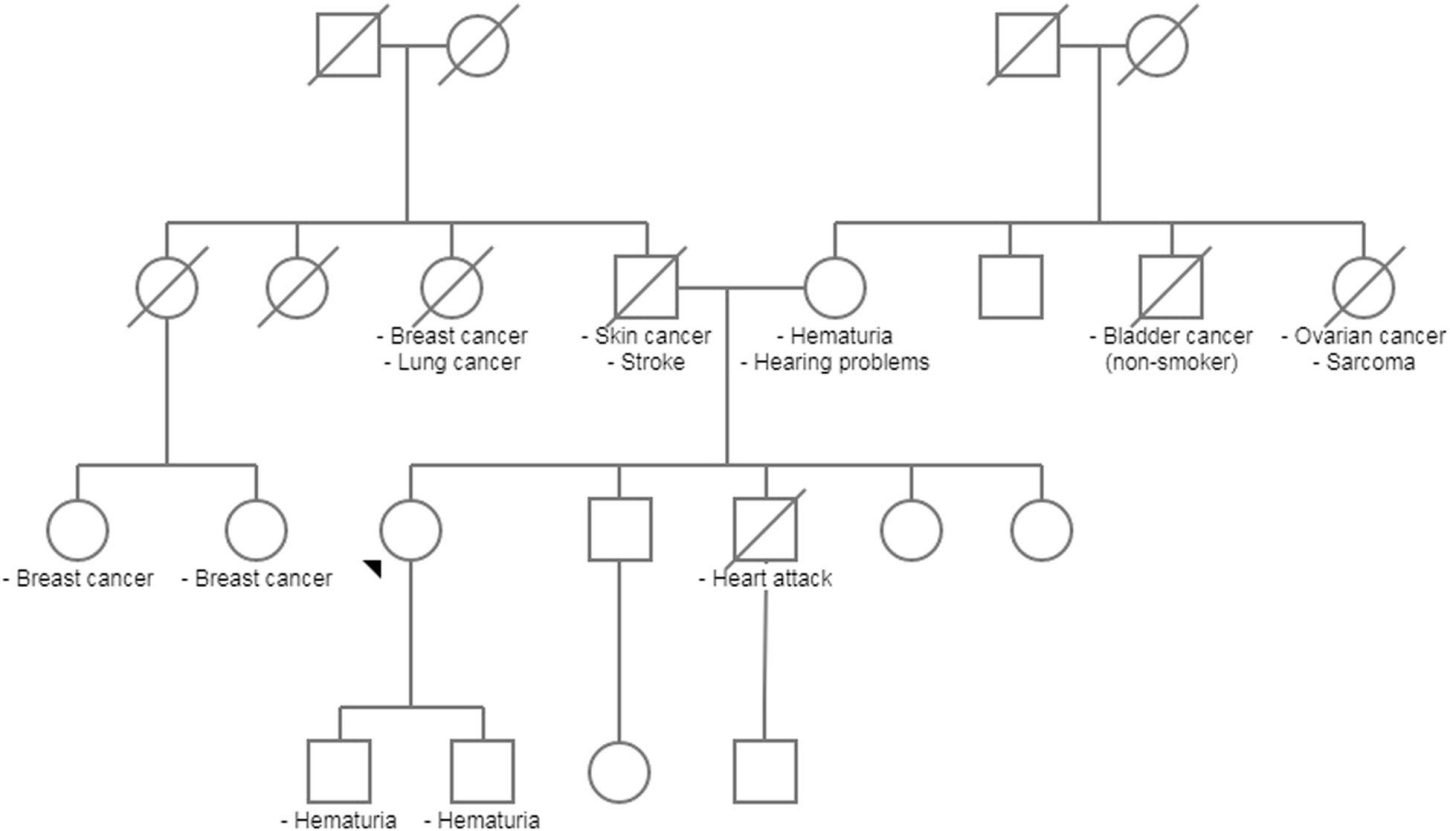
Family 2 pedigree. The proband is indicated with an arrow head

Our second proband is a 48-year-old female with stage IV chronic kidney disease (estimated GFR of 22.5 mL/min), nephrotic syndrome (6 g of protein/24-h urine) and hypertension. She underwent a renal biopsy, which revealed FSGS with patchy moderate tubular atrophy, interstitial fibrosis and mild chronic inflammation. She was initially started on prednisone but did not tolerate it due to excessive weight gain of up to 60 pounds and dyspnea. Her renal condition was treated with a combination of ACE inhibitor and ARB medications and excellent blood pressure control, which decreased her proteinuria to 250 mg/24-h urine. Subsequently, her proteinuria started increasing again, and she was started on tacrolimus but showed poor response. She has a history of endometrial adenocarcinoma and endometrioid ovarian carcinoma. The patient also has a history of hypertension and obesity, as well as a family history of cancer (Fig. 2). The proband’s mother and two sons have hematuria. The patient’s sister carried a diagnosis of FSGS and stage IV chronic kidney disease (estimated GFR of 24 mL/min); her brother passed away at age 49 due to cardiac disease and was noted to have had hearing loss at a young age. Our proband was tested with a nephrotic syndrome/focal segmental glomerulosclerosis sequencing panel (Table 1). She was found to have a heterozygous, likely pathogenic variant in *COL4A4* (c.2842G>T).

**Table 1:** Nephrotic syndrome/focal segmental glomerulosclerosis sequencing panel

Gene	OMIM ID
ACTN4	604638
ANLN	616027
ARHGAP24	610586
ARHGDIA	601925
CD2AP	604241
COL4A3	120070
COL4A4	120131
COL4A5	303630
COL4A6	303631
COQ2	609825
COQ6	614647
COQ8B	615567
CRB2	609720
CUBN	602997
DGKE	601440
EMP2	602334
FAT1	600976
INF2	610982
ITGA3	605025
ITGB4	147557
KANK1	607704
KANK2	614610
KANK4	614612
LAMB2	150325
LMX1B	602575
MYO1E	601479
NEIL1	608844
NEXMIF	300524
NPHS1	602716
NPHS2	604766
NUP107	607617
NUP205	614352
NUP93	614351
PAX2	167409
PDSS2	610564
PLCE1	608414
PTPRO	600579
SCARB2	602257
SMARCAL1	606622
TRPC6	603652
TTC21B	612014
WDR73	616144
WT1	607102
XPO5	607845

## Discussion

Type IV collagen-related nephropathy, in which there is disruption of the normal glomerular basement membrane architecture and kidney disease, has been linked to pathogenic variants in *COL4A3*, *COL4A4*, and *COL4A5*^9^. Heterozygous mutations in *COL4A3* and *COL4A4* result in mild disease with or without isolated microscopic hematuria (MH) characterized by focal or diffuse thinning of the GBM, which is called thin basement membrane nephropathy (TBMN)^9^. Males hemizygous for *COL4A5* pathogenic variants, and individuals of either sex with homozygous or compound heterozygous *COL4A3/4* pathogenic variants, are at a greater risk of developing X-linked and autosomal recessive Alport syndrome, respectively^10^. ESRD develops within the first three decades of life in AS, which is also associated with sensorineural deafness and ocular abnormalities, including asymptomatic dot and fleck retinopathy and lenticonus^11,12^. Females with heterozygous pathogenic variants in COL4A5 can present a variety of clinical features ranging from asymptomatic hematuria to more severe disease with progression to ESRD^13^. Moreover, pathogenic mutations in *COL4A5* have been identified in families with familial and sporadic FSGS^14^. The presence of bilateral renal cysts in our patient is interesting, as, to the best of our knowledge, an association between *COL4A5* variants and renal cysts has not been reported before.

In our first proband, WES detected a heterozygous variant in *COL4A5* (c.141+1G>A, IVS2+1G>A). To the best of our knowledge, this variant has not been reported previously as either a pathogenic or a benign variant. This splice site variant destroys the canonical splice donor site in intron 2 and is predicted to cause abnormal gene splicing. The c.141+1G>A variant has not been not observed in large population cohorts^15–17^. Based on the ACMG 2015 guidelines, the c c.141+1G>A, IVS2+1G>A variant was classified as a pathogenic variant^18^. Even though our second proband had diffuse foot process effacement, her poor response to immunosuppression raised suspicion of a genetic etiology^19^. She was tested with a nephrotic syndrome/focal segmental glomerulosclerosis sequencing panel, which detected a heterozygous and likely pathogenic variant in *COL4A4* (c.2842G>T). This variant is predicted to result in a premature protein termination (p.Gly948). This variant alone is probably sufficient to be the primary cause of the renal disease in this patient. Notably, heterozygous missense variants in *COL4A4* and *COL4A3* have been reported to cause familial or sporadic FSGS^18,20^.

While the first patient underwent WES, which detected a pathogenic variant in COL4A5, the second patient had a nephrotic syndrome/focal segmental glomerulosclerosis sequencing panel that revealed a likely pathogenic variant in COL4A4. There is an ongoing debate between the advantages and drawbacks of each test, especially in Mendelian disorders. One advantage of WES is variant segregation analysis and the concurrent testing of other family members, which can identify the pattern of Mendelian inheritance and detect other incidental variants that can be of clinical significance. Our first proband, thanks to WES, was found to be compound heterozygous for two ABCA4 variants. This finding helps in explaining her macular degeneration and vision loss and would be of great value when counseling this patient and her family. Gene panels have historically been preferred, especially when looking for genes related to a patient’s phenotype, because of their low cost and short turnaround time, and many clinical geneticists consider them a rapid first-tier test.

Molecular genetic testing is noninvasive, can be highly accurate and is becoming the diagnostic procedure of choice for Alport syndrome since renal pathology findings of FSGS do not necessarily indicate genetic mutation^21^. Therefore, a multidisciplinary team approach, with expertize in clinical genetics, nephrology, and nephropathology, is important to attain an accurate diagnosis. While the rate of progression of renal disease may be related to the underlying causal mutation, molecular analysis may eventually provide more prognostic data than either renal or skin biopsy. Massively parallel (next generation) sequencing allows simultaneous analysis of the *COL4A3*, *COL4A4*, and *COL4A5* genes, providing benefits in screening time and cost, and should be considered in similar cases to ours^22,23^. In summary, we present two families with novel pathogenic variants in COL4A5 and COL4A4 diagnosed by exome sequencing and gene panel testing, respectively, and discuss the merits of these approaches.
