# Correction: Novel variants in COL4A4 and COL4A5 are rare causes of FSGS in two unrelated families

**DOI:** 10.1038/s41439-018-0025-7

**Published:** 2018-08-28

**Authors:** Stephanie L. Hines, Anjali Agarwal, Mohamedanwar Ghandour, Nabeel Aslam, Ahmed N. Mohammad, Paldeep S. Atwal

**Affiliations:** 1 Department of Internal Medicine, Mayo Clinic, Jacksonville, FL 32224 USA; 2 Department of Clinical Genomics, Mayo Clinic, Jacksonville, FL 32224 USA

**Correction to:** *Human Genome Variation* **5**, 15 (2018); 10.1038/s41439-018-0016-8; published online 10 July 2018

The originally published version of this article contained an error in the name of the author Nabeel Aslam, which was incorrectly given as Aslam Nabeel. This has now been corrected in both the PDF and HTML versions of the article.

